# A systematic review and meta-analysis of thoracic endovascular aortic repair with the proximal landing zone 0

**DOI:** 10.3389/fcvm.2023.1034354

**Published:** 2023-02-24

**Authors:** Longtu Zhu, Xiaoye Li, Qingsheng Lu

**Affiliations:** Division of Vascular Surgery, Department of General Surgery, Changhai Hospital, Naval Medical University, Shanghai, China

**Keywords:** zone 0 TEVAR, fenestrated TEVAR, chimney TEVAR, hybrid endovascular aortic repair, endograft, complications after TEVAR

## Abstract

**Background:**

Thoracic endovascular aortic repair, initially intended for thoracic aortic disease treatment, has extended its application to the proximal zone of the aorta. However, the safety and surgical outcomes of extending the proximal landing zone into the ascending aorta (zone 0) in selected cases remain unknown. Thus, we performed a systematic review and meta-analysis of zone 0 thoracic endovascular aortic repair (TEVAR) to obtain a deeper understanding of its safety, outcomes, and trends over time.

**Methods:**

A literature search was performed using PubMed, EMBASE, and Web of Science databases in accordance with the preferred reporting items for systematic reviews and meta-analyses guidelines, from January, 1997 to January, 2022. Only studies involving zone 0 TEVAR were included. The retrieved data from the eligible studies included basic study characteristics, 30-day/in-hospital mortality rate, indications, comorbidities, stent grafts, techniques, and complications. Summary effect measures of the primary outcomes were obtained by logarithmically pooling the data with an inverse variance-weighted fixed-effects model.

**Results:**

Fifty-three studies with 1,013 patients were eligible for analysis. The pooled 30-day/in-hospital mortality rate of zone 0 TEVAR was 7.49%. The rates of post-operative stroke, type Ia endoleak, retrograde type A aortic dissection, and spinal cord ischemia were 8.95, 9.01, 5.72, and 4.12%, respectively.

**Conclusions:**

Although many novel stent grafts and techniques targeting zone 0 TEVAR are being investigated, a consensus on technique and device selection in zone 0 TEVAR is yet to be established in current practice. Furthermore, the post-operative stroke rate is relatively high, while other complication rates and perioperative death rate are comparable to those of TEVAR for other aortic zones.

## 1. Introduction

Thoracic endovascular aortic repair (TEVAR) has become a viable treatment option for thoracic aortic pathologies over the past years (1, 2). The proximal landing zone (PLZ) of the stent graft has been extended from the descending to the ascending aorta (zone 0) to ensure a sufficient and healthy PLZ. Following the development of surgical devices and improvement in supra-aortic vessels revascularization techniques, studies investigating the feasibility and safety of TEVAR with zone 0 landing have been conducted.

Unlike TEVAR for other aortic zones, zone 0 TEVAR lacks high-quality evidence to support its use. Studies on zone 0 TEVAR mostly comprise case reports, case series, and retrospective studies. As no standard off-the-shelf stent grafts dedicated to zone 0 TEVAR are available, the safety, feasibility, and efficacy of zone 0 TEVAR with off-label use of thoracic or custom-made stent grafts are yet to be studied (3). Thus, a timely and comprehensive understanding of the safety and outcomes of zone 0 TEVAR is necessary before further promotion of its application. This systematic review and meta-analysis aimed to provide a comprehensive overview of the current application of zone 0 TEVAR, including its indications, stent grafts, procedures, and post-operative complications.

## 2. Methods

### 2.1. Search methodology

The systematic review conformed to the preferred reporting items for systematic review and meta-analyses statement standards (4). A search in PubMed, EMBASE, and Web of Science databases from January, 1997 to January, 2022 was made using set algorithms. The search algorithm in PubMed was “((((landing zone) AND (ascending aorta)) OR (‘landing' [All Fields] AND ‘zone' [All Fields] AND (‘zone' [All Fields] AND ‘0' [All Fields]))) OR (‘stent graft' [All Fields] AND (‘zone' [All Fields] AND ‘0' [All Fields]))) OR (‘endovascular' [All Fields] AND (‘zone' [All Fields] AND ‘0' [All Fields])).” The search algorithm used for EMBASE was “1. TEVAR; 2. Zone 0; 3. Ascending aorta; 4. 2 OR 3; 5. 1 AND 4.” The search algorithm in Web of Science was “((TS = (zone 0)) OR TS = (ascending aorta)) AND TS = (TEVAR).”

### 2.2. Inclusion and exclusion criteria

The studies included were published in English, containing zone 0 TEVAR, and clinical studies or cohort case reports. Studies containing only zone 0 TEVAR with prosthetic ascending aorta as the PLZ and studies, in which the reported number of cases of zone 0 TEVAR were <5, were excluded from the analysis.

### 2.3. Data extraction

Two researchers independently extracted data. The authors, publication date, study region, research type, number of cases, recruitment time, sex, age, 30-day/in-hospital mortality, 30-day/in-hospital mortality rate, pathology, stent graft, technique, and complications were retrieved from the eligible studies.

### 2.4. Statistical analysis

Data on stent graft, procedure and complications were summarized. Excel software (Microsoft Corp, Redmond, WA, USA) and Review Management 5.4 (The *Cochrane*. *Collaboration*, Oxford, UK) were used to record, analyze, conduct the meta-analysis, and tabulate clinical data. A meta-analysis was performed for perioperative mortality of zone 0 TEVAR and post-operative stroke, type Ia endoleak, spinal cord ischemia (SCI), and retrograde type A dissection (RTAD). Summary effect measures of post-operative complications and perioperative death rates were obtained by logarithmically pooling the data with an inverse variance-weighted fixed-effects model and presented with a 95% confidence interval (CI). Heterogeneity of the summary effects measures was assessed with the *I*^2^ test and considered heterogeneous when *I*^2^ was >50%. A 2-sided *P*-value of <0.05 was considered statistically significant.

## 3. Results

### 3.1. Study selection

**Figure 1:**
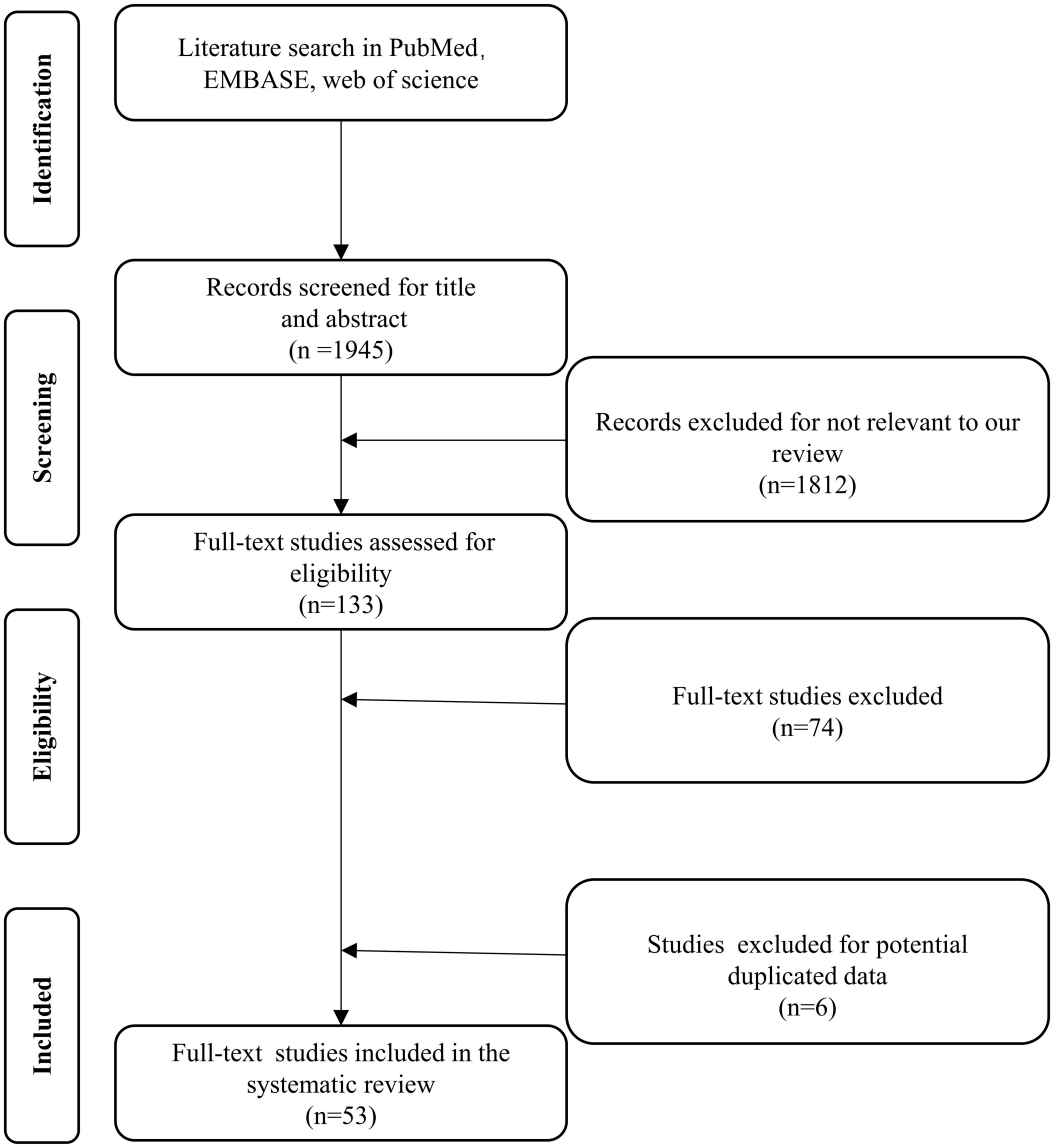
Preferred reporting items for systematic reviews and meta-analysis flow chart.

A total of 1,812 studies were retrieved from PubMed, EMBASE, and Web of Science between January, 1997 and January, 2022. A total of 133 studies were considered eligible according to the inclusion criteria, of which 74 were excluded according to the exclusion criteria (Figure 1). Six studies were excluded because they were duplicates. Fifty-three studies, with a total number of 1,013 cases, were included in the final analysis (Table 1). The present study included 48 retrospective and 5 prospective studies.

**Table 1:** Detailed information in each manuscript (*N* = 1,013).

**References**	**Publish date**	**Cases of zone 0 TEVAR**	**Recruitment period**	**Female**	**Male**	**Mean age**	**30-day/in-hospital death**	**30-day/in-hospital death rate**
Kurimoto et al. (5)	2009/5/1	23	2001–2008	N/A	N/A	N/A	N/A	N/A
Chiesa et al. (6)	2010/2/1	24	1999–2009	3	21	73	3	12.50%
Holt et al. (7)	2010/6/1	9	2001–2009	3	6	64	1	11.11%
Geisbüsch et al. (8)	2010/6/1	10	1997–2009	2	8	65	1	10.00%
Canaud et al. (9)	2010/7/1	6	1998–2008	N/A	N/A	N/A	0	0.00%
Kolvenbach et al. (10)	2011/5/1	11	2008–2010	6	5	73	1	9.09%
Vallejo et al. (11)	2012/2/1	27	2002–2010	N/A	N/A	N/A	8	29.63%
Melissano et al. (12)	2012/3/1	32	1999–2011	N/A	N/A	N/A	3	9.38%
Fukui et al. (13)	2013/1/20	9	2007–2012	N/A	N/A	N/A	N/A	N/A
Preventza et al. (14)	2013/9/1	29	2005–2011	8	21	67	2	6.90%
Shirakawa et al. (15)	2014/2/1	30	1997–2012	1	21	74	1	3.33%
Bernardes et al. (16)	2014/7/1	7	2007–2012	4	3	59	1	14.29%
Roselli et al. (17)	2015/1/1	22	2006–2014	11	11	72	3	13.64%
Hiraoka et al. (18)	2015/1/1	7	2005–2013	N/A	N/A	N/A	5	71.43%
De Rango et al. (19)	2014/1/1	19	2005–2013	N/A	N/A	N/A	3	15.79%
Kurimoto et al. (20)	2015/7/1	37	2007–2013	8	29	78	0	0.00%
Gandet et al. (21)	2015/7/1	13	2001–2013	2	13	74	N/A	N/A
Cazavet et al. (22)	2016/1/1	17	2002–2014	N/A	N/A	N/A	N/A	N/A
Katada et al. (23)	2016/2/1	7	2012–2014	2	5	73	0	0.00%
Ziza et al. (24)	2016/6/1	17	1998–2013	N/A	N/A	N/A	3	17.65%
Tsilimparis et al. (25)	2016/6/1	10	2011–2014	5	5	67	0	0.00%
Böckler et al. (26)	2016/6/1	7	2009–2010	N/A	N/A	N/A	1	14.29%
Narita et al. (27)	2016/7/1	35	2008–2014	5	30	79	2	5.71%
Faure et al. (28)	2016/7/1	11	2005–2015	N/A	N/A	N/A	N/A	N/A
Yoshitake et al. (29)	2016/10/1	23	2011–2015	3	20	76	1	4.35%
Pecoraro et al. (30)	2017/6/1	26	2006–2015	9	17	72	2	7.69%
Canaud et al. (31)	2017/8/1	16	2013–2016	N/A	N/A	75	N/A	N/A
Wang et al. (32)	2017/10/1	22	2009–2016	2	20	61	0	0.00%
Roselli et al. (33)	2018/4/1	39	2006–2016	16	23	72	5	12.82%
Toya et al. (34)	2018/11/1	8	2015–2016	3	5	73	0	0.00%
Zhu et al. (35)	2019/1/1	5	2015–2017	N/A	N/A	N/A	N/A	N/A
Hosaka et al. (36)	2019/1/1	22	2009–2013	N/A	N/A	N/A	0	0.00%
Huang et al. (37)	2019/1/20	22	2012–2017	0	22	54	1	4.55%
Yamauchi et al. (38)	2019/3/1	7	2012–2017	N/A	N/A	N/A	N/A	N/A
Ryomoto et al. (39)	2019/8/1	9	2010–2017	N/A	N/A	N/A	N/A	N/A
Piffaretti et al. (40)	2019/11/1	6	2011–2015	3	3	69	N/A	N/A
Tsilimparis et al. (41)	2020/5/1	12	2011–2017	N/A	N/A	N/A	1	8.33%
De León et al. (42)	2020/6/27	60	2007–2015	N/A	N/A	N/A	N/A	N/A
Kuo et al. (43)	2020/9/1	13	2016–2017	4	9	64	0	0.00%
Tinelli et al. (44)	2020/10/1	6	2009–2018	N/A	N/A	N/A	1	16.67%
Fernández-Alonso et al. (45)	2020/11/1	6	2014–2020	N/A	N/A	N/A	1	16.67%
Chassin-Trubert et al. (46)	2021/2/1	42	2004–2018	7	35	70	6	14.29%
Li et al. (47)	2021/3/1	43	2015–2019	14	29	64	0	0.00%
Planer et al. (48)	2021/3/4	28	N/A	6	22	72	2	7.14%
Dake et al. (49)	2021/6/1	8	N/A	1	7	73	2	25.00%
Seguchi et al. (50)	2021/6/25	7	2016–2019	1	6	83	0	0.00%
Li et al. (51)	2021/8/1	16	2009–2011	0	16	55	1	6.25%
Li et al. (52)	2021/8/24	37	2016–2019	7	30	70	2	5.41%
Hanna et al. (53)	2021/11/1	6	2009–2019	N/A	N/A	N/A	N/A	N/A
Barnes et al. (54)	2021/11/12	6	2011–2019	N/A	N/A	N/A	1	16.67%
Kudo et al. (55)	2021/11/20	40	2010–2020	12	28	79	1	2.50%
Chen et al. (56)	2021/12/29	51	2010–2019	N/A	N/A	N/A	0	0.00%
Eleshra et al. (57)	2022/1/31	8	2012–2016	1	7	70	1	12.50%

N/A, not available, which means the specific information was not available or could not be extracted from manuscripts; TEVAR, thoracic endovascular aortic repair.

### 3.2. Pathology and comorbidities

The type of aortic pathologies and comorbidities are summarized in Tables 2, 3, respectively. Indications for zone 0 TEVAR of 636 cases from 32 studies were disclosed, and included aneurysm (*n* = 347, 54.56%), aortic dissection (*n* = 214, 33.65%), intramural hematoma (*n* = 56, 8.81%), penetrating atherosclerotic ulcer (*n* = 10, 1.57%), Kommerell's diverticulum (*n* = 6, 0.94%), and traumatic aortic rupture (*n* = 3, 0.47%).

**Table 2:** Aortic pathology in included studies (*N* = 636).

**Pathologies**	**No. of cases (** ***n*** **, %)**	
Aneurysm	347	54.56%
Dissection	214	33.65%
IMH	56	8.81%
PAU	10	1.57%
Kommerell's diverticulum	6	0.94%
Traumatic rupture of aorta	3	0.47%

PAU, penetrating atherosclerotic ulcer; IMH, intramural hematoma.

**Table 3:** Comorbidities in included studies.

**Comorbidities**	**No. of cases (** ***n*** **, %)**	
Smoking	164/373	43.97%
Diabetes	65/396	16.41%
Hypertension	352/442	79.64%
ASA > II	69/106	65.09%
CHF	25/147	17.01%
COPD	114/401	28.43%
Renal insufficiency	73/521	14.01%
CVD	99/545	18.17%
Dyslipidemia	90/213	42.25%
Peripheral vascular occlusive disease	44/217	20.28%
End-stage renal disease	5/77	6.49%
CAD	149/452	32.96%
Concomitant malignancy	15/56	26.79%
Connective tissue diseases	6/68	8.82%

ASA, American Society of Anesthesiologists classification; CHF, congestive heart failure; COPD, chronic obstructive pulmonary disease; CVD, cerebrovascular diseases; CAD, coronary artery disease.

### 3.3. Stent graft

Stent grafts used in zone 0 TEVAR are summarized in Table 4. A total of 554 stent grafts from 25 studies were analyzed. The most frequently used stent graft in zone 0 TEVAR was TAG/c-TAG stent graft (W.L. Gore and Associates, AZ, USA) (*n* = 215, 38.81%) followed by Valiant thoracic stent graft (Medtronic, MN, USA) (*n* = 83, 14.98%), Zenith TX1/TX2 (Cook Medical, IN, USA) (*n* = 58, 12.27%), Najuta (Kawasumi Laboratories, Tokyo, Japan) (*n* = 68, 12.27%), RELAY endovascular thoracic stent graft (Terumo Aortic, FL, USA) (*n* = 34, 6.14%), Ankura thoracic stent graft (Lifetech Scientific, Shenzhen, China) (*n* = 30, 5.42%), *NEXUS* Aortic Arch *Stent* Graft System (Endospan, Herzlia, Israel) (*n* = 29, 5.32%), Castor (Microport Medical, Shanghai, China) (*n* = 18, 3.25%), Thoracic Branch Endoprosthesis (Gore & Associates, AZ, USA) (*n* = 8, 1.44%), Zenith ascending stent graft (Cook Medical, IN, USA) (*n* = 10, 1.81%), and E-vita (Jotec, Hechingen, Germany) (*n* = 1, 0.18%). In 337 (60.83%) cases, stent grafts had a proximal bare-metal portion. Three novel stent grafts dedicated to zone 0 TEVAR had been introduced in the reviewed studies (25, 34, 48). Zenith ascending stent graft is aimed at the ascending aorta and has no branches or fenestrations for supra-aortic vessels. Najuta thoracic stent graft system is a fenestrated stent graft with one to three fenestrations, which can preserve all supra-aortic vessels. *NEXUS* Aortic Arch *Stent* Graft System is a novel single branch, two stent graft system used for endovascular aortic arch repair.

**Table 4:** Stent graft category (*N* = 554).

**Devices**	**No. of stent grafts (** ***n*** **, %)**	
TAG/cTAG stent graft (Gore & Associates, AZ, USA)	215	38.81%
Valiant thoracic stent graft (Medtronic, MN, USA)	83	14.98%
Zenith TX1/TX2 (Cook Medical, IN, USA)	58	10.47%
Najuta thoracic stent graft system (Kawasumi Laboratories, Tokyo, Japan)	68	12.27%
RELAYendovascular thoracic stent graft (Terumo Aortic, FL, USA)	34	6.14%
Ankura thoracic stent graft (Lifetech Scientific, Shenzhen, China)	30	5.42%
NEXUS Aortic Arch Stent Graft System (Endospan, Herzlia, Israle)	29	5.32%
Castorstent (Microport Medical, Shanghai, China)	18	3.25%
Zenith ascending stent graft (Cook Medical, IN, USA)	10	1.81%
Thoracic Branch Endoprosthesis (Gore & Associates, AZ, USA)	8	1.44%
E-vita OPEN NEO hybrid stent graft system (Jotec, Hechingen, Germany)	1	0.18%

### 3.4. Procedure

**Figure 2:**
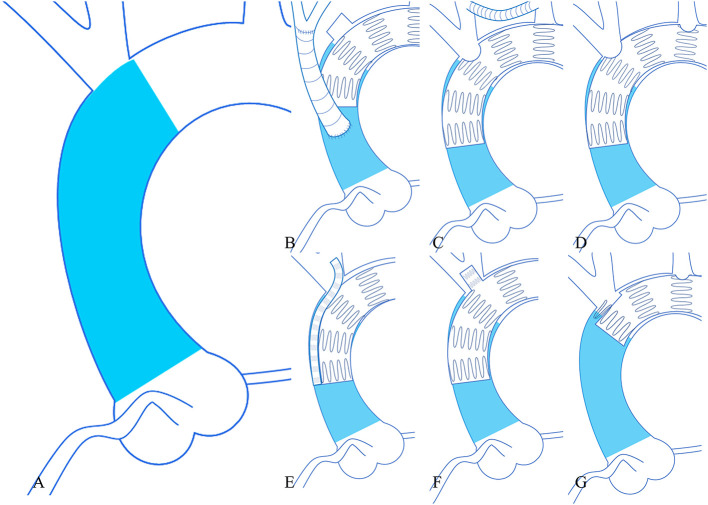
Six techniques used in zone 0 TEVAR. **(A)** (Left larger image) the area of zone 0 (blue). **(B)** (Upper left) TEVAR + debranching procedure. **(C)** (Upper middle) TEVAR + bypass procedure. **(D)** (Upper right) fenestrated TEVAR. **(E)** (Lower left) chimney TEVAR. **(F)** (Lower middle) branched TEVAR. **(G)** (Lower right) proximal scalloped and fenestrated TEVAR. TEVAR, thoracic endovascular aortic repair.

The procedures performed in 783 cases from 46 studies were classified as following: 1. TEVAR without any modification or parallel stent technique + bypass/debranching of supraaortic vessels (*n* = 384, 49.04%); 2. TEVAR + chimney + bypass/debranching of supraaortic vessels (*n* = 75, 9.58%); 3. fenestrated TEVAR + bypass/debranching of supraaortic vessels (*n* = 28, 3.58%); 4. branched TEVAR + bypass/debranching of supraaortic vessels (*n* = 63, 8.05%); 5. proximal scalloped TEVAR + bypass/debranching of supraaortic vessels (*n* = 15, 1.92%); 6. TEVAR only in ascending aorta (*n* = 20, 2.55%); 7. chimney TEVAR (*n* = 24, 3.07%); 8. fenestrated TEVAR (*n* = 163, 20.82%); and 9. proximal scalloped and fenestrated TEVAR (*n* = 11, 1.40%) (Table 5). Figure 2 illustrates the techniques used in zone 0 TEVAR. Out of the 783 cases, the inflow of the left subclavian artery (LSA) was preserved in 642 (81.99%) cases. Among the 191 cases treated with fenestrated TEVAR, pre-operative fenestrations were used in 44 (23.04%), back table fenestrations in 93 (48.69%), laser-*in situ* fenestrations in 43 (22.51%), and needle-*in situ* fenestrations in 9 (4.71%) cases, while fenestration techniques were not disclosed in 2 (1.05%) cases.

**Table 5:** Surgery details and LSA information (*N* = 783).

**Surgeries**	**No. of cases (*****n*** **%)**		Preservation of LSA inflow^*^	**No. of cases (*****n*** **%)**	
TEVAR without any modification or parallel stent technique + bypass/transposition of supra-aortic vessels	384	49.04%	Y	318	82.81%
			N	66	17.19%
TEVAR+chimney + bypass/debranching of supra-aortic vessels	75	9.58%	Y	71	94.67%
			N	4	5.33%
Fenestrated TEVAR + bypass/debranching of supra-aortic vessels	28	3.58%	Y	23	82.14%
			N	5	17.86%
Branched TEVAR + bypass/debranching of supra-aortic vessels	63	8.05%	Y	60	95.24%
			N	3	4.76%
Proximal scalloped TEVAR + bypass/debranching of supra-aortic vessels	15	1.92%	Y	14	93.33%
			N	1	6.67%
TEVAR only in ascending aorta	20	2.55%	Y	20	100.00%
			N	0	0.00%
Chimney TEVAR	24	3.07%	Y	5	20.83%
			N	19	79.17%
Fenestrated TEVAR	163	20.82%	Y	147	90.18%
			N	16	9.82%
Proximal scalloped and fenestrated TEVAR	11	1.40%	Y	6	54.55%
			N	5	45.45%

^*^The only intended covered supraarch artery without revascularization was LSA, when other branch arteries were invariably revascularized. LSA, left subclavian artery; TEVAR, thoracic endovascular aortic repair.

### 3.5. Perioperative mortality and post-operative complications

**Figure 3:**
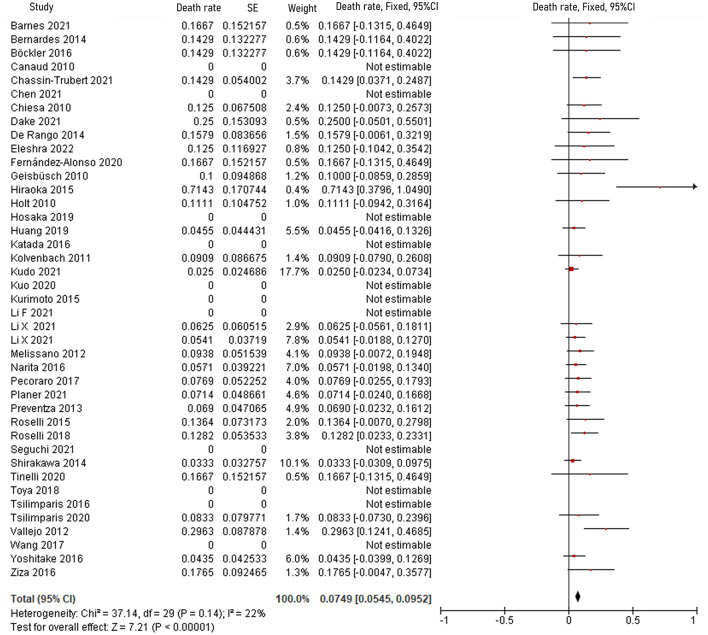
Forest plot shows the fixed-effects proportion meta-analysis for perioperative death rate. CI, confidence interval; SE, standard error.

**Figure 4:**
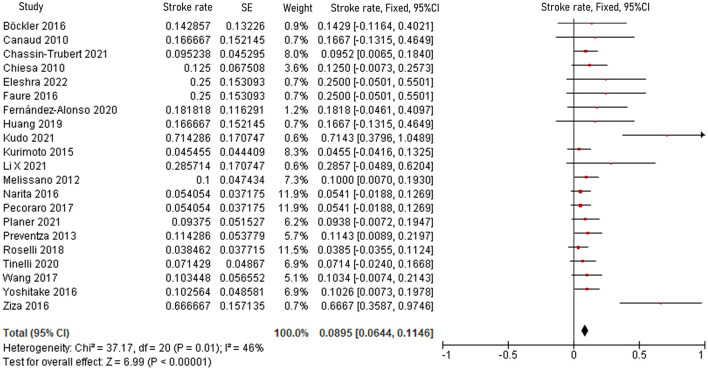
Forest plot showing the fixed-effects proportion meta-analysis for post-operative stroke rate. CI, confidence interval; SE, standard error.

**Figure 5:**
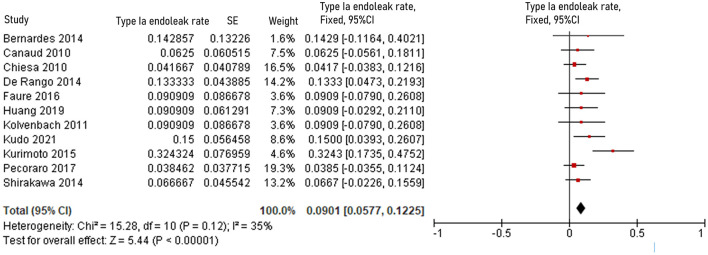
Forest plot showing the fixed-effects proportion meta-analysis for type Ia endoleak rate. CI, confidence interval; SE, standard error.

**Figure 6:**
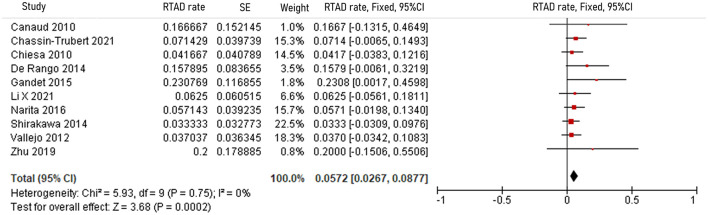
Forest plot showing the fixed-effects proportion meta-analysis for RTAD rate. CI, confidence interval; SE, standard error; RTAD, retrograde type A dissection.

**Figure 7:**
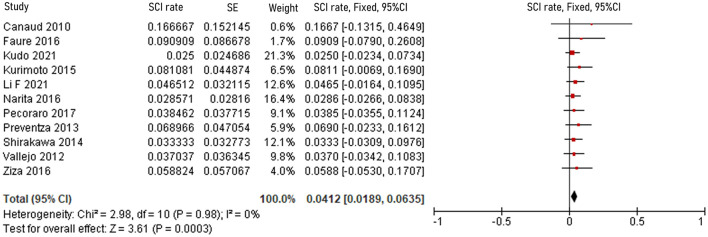
Forest plot showing the fixed-effects proportion meta-analysis for RTAD rate. CI, confidence interval; SE, standard error; RTAD, retrograde type A dissection.

The pooled 30-day/in-hospital death rate was 7.49% (95% CI, 5.45–9.52, *P* < 0.00001, *I*^2^ = 22%, Figure 3). Data on the causes, characteristics, and outcomes of stroke, SCI, type Ia endoleak, and RTAD were collected, and the incidences were obtained by logarithmically pooling the data with an inverse variance-weighted fixed-effects model. The most common post-operative complication was stroke (8.95%, 95% CI, 6.44–11.46, *P* < 0.00001, *I*^2^ = 46%, Figure 4), followed by type Ia endoleak (9.01%, 95% CI, 5.77–12.25, *P* < 0.00001, *I*^2^ = 35%, Figure 5), RTAD (5.72%, 95% CI, 2.67–8.77, *P* = 0.0002, *I*^2^ = 0%, Figure 6), and SCI (4.12%, 95% CI, 1.89–6.35, *P* = 0.0003, *I*^2^ = 0%, Figure 7).

The causes and outcomes of stroke in 26 studies are presented in Table 6. Causes were disclosed in 11 studies, and outcomes were disclosed in 16 studies. Atherosclerotic plaque was considered the principal cause of stroke in six studies (12, 18, 26, 29, 45, 55). Other possible causes suggested by the authors included migration and compression of chimney stents, LSA dissection, debranching of LSA, and lower left ventricular ejection fraction (27, 32, 39, 44). Based on available data, it appears that stroke was the cause of death in 12 of the 65 patients who suffered from this complication (6, 14, 18, 26, 37, 44, 45).

**Table 6:** Possible causes and outcomes of strokes in included studies.

**References**	**Stroke cases/total cases in the articles %**		**Possible causes**	**Outcomes**
Pecoraro et al. (30)	1/26^a^	3.85%	Not available	1 recovered spontaneously
Huang et al. (37)	1/22^a^	4.55%	Not available	1 dead due to stroke
Kurimoto et al. (20)	2/37^b^	5.41%	Not available	Not available
Li et al. (52)	2/37^a^	5.41%	Not available	1 dead due to cardiac attack 1 dead due to severe pulmonary infection
Planer et al. (48)	2/28^b^	7.14%	Not available	2 recovered with minor sequalae
Wang et al. (32)	2/22^a^	9.09%	Migration of chimney stents Compression of chimney stents	2 recovered by additional stent placement^*^
Melissano et al. (12)	3/32^a^	9.38%	Atherosclerotic plaques in the aortic arch	Not available
Chassin-Trubert et al. (46)	4/42^b^	9.52%	Not available	Not available
Kudo et al. (55)	4/40^a^	10.00%	Atherosclerotic plaques in the aortic arch	Not available
Roselli et al. (33)	4/39^a^	10.26%	Not available	1 dead due to unknown reason
Preventza et al. (14)	3/29^a^	10.34%	Not available	1 deaddue to stroke 2 recovered
Narita et al. (27)	4/35^a^	11.43%	Prolonged procedural time Increased blood loss	2 dead due to unmentioned reason
Chiesa et al. (6)	3/24^a^	12.50%	Not available	3 dead due to stroke
Böckler et al. (26)	1/7^a^	14.29%	Atherosclerotic plaques in the aortic arch	1 dead due to stroke
Canaud et al. (9)	1/6^a^	16.67%	Not available	Not available
Fernández-Alonso et al. (45)	1/6^a^	16.67%	Atherosclerotic plaques in the aortic arch	1 dead due to stroke
Tinelli et al. (44)	1/6^a^	16.67%	LSA dissection	1 dead due to stroke
Yoshitake et al. (29)	4/23^a^	17.39%	Atherosclerotic plaques	3 dead due to COPD, cancer, pneumonia
Ziza et al. (24)	3/17^a^	17.65%	Supra-arch vessels dissection	Not available
Faure et al. (28)	2/11^a^	18.18%	Not available	Not available
Eleshra et al. (57)	2/8^a^	25.00%	Not available	Not available
Dake et al. (49)	2/8^b^	25.00%	Not available	1 dead due to other pathologies
Katada et al. (23)	2/7^a^	28.57%	Not available	2 sustaining grade III focal neurologic deficits
Hiraoka et al. (18)	5/7^a^	71.43%	Atherosclerotic plaques in the aortic arch	4 dead due to stroke
Ryomoto et al. (39)	6/9^a^	66.67%	Prolonged procedural time Lower left ventricular ejection fraction	Not available

^*a*^Perioperative (30-day) stroke.

^*b*^Late (≥30-day) stroke.

^*^The stroke was discovered immediately after operation. LSA, left subclavian artery; COPD, chronic obstructive pulmonary diseases.

Causes and outcomes of type Ia endoleak in 11 studies are shown in Table 7. Causes were disclosed in seven studies, while outcomes were disclosed in eight studies. Migration of stent graft was considered the principal cause of type Ia endoleak in three studies (16, 28, 37). Other possible causes suggested by the authors included aneurysmal evolution of aorta, unfavorable stent placement, gutter leakage between the main stent graft and chimney stent, short distance between the ostium of innominate artery and the aneurysm, lack of comfortability of Najuta stent prototype, and short proximal neck length (10, 20, 31, 37, 55). Based on available data, 17 of the 36 patients who presented with type Ia endoleak were treated conservatively, while in 5 patients type Ia endoleak lead to aneurysm enlargement, which ruptured and caused death in 1 patient (6, 10, 15, 20, 28, 30, 42).

**Table 7:** Possible causes and outcomes of type Ia endoleaks in included studies.

**References**	**Type Ia endoleak cases/Total casesin the articles %**		**Possible causes**	**Outcomes**
Pecoraro et al. (30)	1/26^a^	3.85%	Not available	1 under surveillance without reintervention
Chiesa et al. (6)	1/24^a^	4.17%	Not available	Resolved spontaneously
Canaud et al. (9)	1/16^b^	6.25%	Aneurysmal evolution of aorta	1 dead due to aneurysm rupture
Shirakawa et al. (15)	2/30^a^	6.67%	Not available	2 under surveillance without reintervention
Kolvenbach et al. (10)	1/11^a^	9.09%	Unfavorable stent placement	1 under surveillance without reintervention
Faure et al. (28)	1/11^a^	9.09%	Migration of stentgraft	1 under surveillance without reintervention
Huang et al. (37)	2/22^a^	9.09%	Migration of stentgraft Aneurysmal evolution of aorta	Not available
De León et al. (42)	8/60^a^	13.33%	Not available	6 recovered spontaneously 2 resolved surgically
Bernardes et al. (16)	1/7^a^	14.29%	Migration of stentgraft	Not available
Kudo et al. (55)	6/40^a^	15.00%	Gutter leakage between the main stent graft and chimney ste Short distance between the ostium of innominate artery and the aneurysm	Not available
Kurimoto et al. (20)	12/37^a^	32.43%	Lack of comfortability of Najuta stent prototype Short proximal neck length	4 resolved by undergoing reinterventions because of aneurysm enlargement 2 dead due to pneumonia 6 under surveillance without reintervention

^*a*^Perioperative (30-day) type Ia endoleak.

^*b*^Late (≥30-day) type Ia endoleak.

Tables 8, 9 show the causes and outcomes of SCI and RTAD in 11 and 9 studies, respectively. One study suggested that SCI might have been caused by LSA coverage and long extent of aortic coverage (20). Outcomes of SCI were disclosed in 10 studies. Three out of 15 SCI patient were left with long-term sequelae (20, 30). Causes of RTAD were disclosed in seven studies, and outcomes were disclosed in nine studies, with clamp injury in hybrid procedure being considered the principal cause in four manuscripts (9, 21, 27, 46). An acute angle formed by the ascending aorta and PLZ, lack of conformability of the COOK TX2 stent graft in zone 0, primary disease progression, >30% oversizing of the stent graft, angulation of the proximal aortic arch >120° and stent graft diameter >42 mm have also been suggested as possible causes of RTAD (21, 35, 51). Four patients died of complications related to RTAD, and based on the available data eight out of 17 patients received reintervention (6, 9, 15, 19, 21, 46).

**Table 8:** Possible causes and outcomes of SCIs in included studies.

**References**	**SCI cases/total cases in the articles %**		**Possible causes**	**Outcomes**
Kudo et al. (55)	1/40^a^	2.50%	Not available	Not available
Narita et al. (27)	1/35^a^	2.86%	Not available	1 recovered after cerebrospinal fluid drainage
Shirakawa et al. (15)	1/30^a^	3.33%	Not available	1 dead due to other reasons
Vallejo et al. (11)	1/27^a^	3.70%	Not available	1 dead due to other reasons
Pecoraro et al. (30)	1/26^a^	3.85%	Not available	1 dead due to respiratory insufficiency and spinal cord ischemia
Li et al. (47)	2/43^a^	4.65%	Not available	2 recovered after cerebrospinal fluid drainage
Ziza et al. (24)	1/17^a^	5.88%	Not available	1 recovered after cerebrospinal fluid drainage
Preventza et al. (14)	2/29^a^	6.90%	Not available	1 recovered after cerebrospinal fluid drainage 1 partial recovered after cerebrospinal fluid drainage
Kurimoto et al. (20)	3/37^a^	8.11%	Long extent of aortic coverage^*^	1 recovered after cerebrospinal fluid drainage 1 permanent paraplegias 1 paraparesis
Faure et al. (28)	1/11^a^	9.09%	Not available	1 recovery after cerebrospinal fluid drainage
Canaud et al. (9)	1/6^a^	16.67%	Not available	1 recovery after cerebrospinal fluid drainage

^*a*^Perioperative (30-day) SCI.

^*^The covered areas of the stent grafts were from ascending aorta to levels of Th 6, Th 8, and Th 10, respectively. SCI, spinal cord ischemia; LSA, left subclavian artery.

**Table 9:** Possible causes and outcomes of RTADs in included studies.

**References**	**RTAD cases/total cases in the articles %**		**Possible causes**	**Outcomes**
Shirakawa et al. (15)	1/30^a^	3.33%	Debranching procedures	1 recovered surgically
Vallejo et al. (11)	1/27^a^	3.70%	Not available	1 treated medically and stable
Chiesa et al. (6)	1//24^b^	4.17%	Not available	1 recovered surgically
Chassin-Trubert et al. (46)	3/42^a^	7.14%	Clamp injury in hybrid procedure	2 immediate ascending aortic replacement 1 dead due to RTAD
Narita et al. (27)	2/35^a^	5.71%	Clamp injury in hybrid procedure	2 managed conservatively
Li et al. (51)	1/16^a^	6.25%	Ascending aorta and proximal landing zone of stent graft in an acute angle	1 dead due to pericardial effusion
De Rango et al. (19)	3/19^a^	15.79%	Not available	2 dead due to RTAD 1 recovered surgically
Canaud et al. (9)	1/6^a^	16.67%	Clamp injury in hybrid procedure	1 immediate ascending aortic replacement
Zhu et al. (35)	1/5^a^	20.00%	Lack of conformability of the COOK TX2 stent graft in zone 0 Primary disease progression	Not available
Gandet et al. (21)	3/13^a^	23.08%	Clamp injury in hybrid procedure Oversizing of stentgraft >30% Angulation of proximal aortic arch >120° Diameter of stentgraft >42 mm	2 recovered surgically 1 dead due to RTAD

^*a*^Perioperative (30-day) RTAD.

^*b*^Late (≥30-day) RTAD. RTAD, retrograde type A dissection.

## 4. Discussion

To meet the challenges posed by the lack of dedicated stent grafts for zone 0 TEVAR, numerous procedures and novel devices have been developed. Techniques range from hybrid surgery to fenestrated, chimney, and branched TEVAR, while newer devices try to provide simpler and safer procedures. According to the reviewed studies, no clear protocol was shown regarding the selection technique or device for zone 0 TEVAR.

Stroke is the most common post-operative complication after zone 0 TEVAR (8.95%). Two meta-analyses of TEVAR for descending thoracic aortic diseases performed by Karaolanis et al. and Allmen et al. suggested that stroke rates in TEVAR for descending aortic aneurysm and type B dissection were 4.1 and 4.4%, respectively, lower than those in zone 0 TEVAR (58, 59). A meta-analysis from 2019 involving 989 patients undergoing total arch replacement with frozen elephant trunk also showed a lower stroke rate (8.95 vs. 2.38%) (60). Six authors suggested detachment of atherosclerotic plaque debris in the aortic arch, induced by manipulation of the guide-wire or stent graft delivery system was the cause of stroke (12, 18, 26, 29, 45, 55). Three authors suggested that the occlusion of supra-aortic vessels owing to migration or compression of stents, and the dissection of supra-aortic vessels could be a further cause (24, 32, 44). Other possible causes included prolonged procedural time, lower left ventricular ejection fraction, and increased blood loss (27, 39). While no author in reviewed studies attributed stroke to planned LSA coverage without revascularization, the meta-analysis made by Chen et al. and Karaolanis et al. reported a significant reduction in stroke rate when the covered LSA had been revascularized (59, 61). However, the stroke rate in the planned LSA coverage without revascularization remains unknown in reviewed studies. To prevent stroke, some authors introduced procedures such as mini-cardiopulmonary bypass support and temporary inflow blockage of branch vessels (39, 50). The principal goal of these interventions is to prevent atheromatous debris from accessing the cerebral blood supply. Given 8.95% stroke rate and 18.46% (12/65) stroke-related death in the review, post-operative stroke prevention must be considered more when planning zone 0 TEVAR (6, 14, 18, 26, 37, 44, 45).

The incidence of type Ia endoleak in zone 0 TEVAR is 9.01% in the review. That is slightly lower than that (10.07%) reported in the multicenter study, involving 1, 18, 43, 55, and 22 cases of zone 0, 1, 2, 3, 4 TEVAR, conducted by Hammo et al. (62) in 2019. In the aortic arch, the varied lengths of the outer and inner curves pose a barrier to the stent graft's stability. This leads a bird-beak configuration after TEVAR increasing the risk of type Ia endoleak. However, Kudo et al. (63) proposed that bird-beak configuration did not occur during the early or late periods after zone 0 TEVAR. Causes suggested by the authors include migration, unfavorable deployment, and lack of flexibility of the stent graft (10, 16, 20, 28, 37). Reinterventions, including open or endovascular surgery, are appropriate treatments for endoleak (20, 42). De León et al. (42) classified type Ia endoleak as “fast” and “slow” based on the time needed to visualize the aneurysmal sac during arteriogram. Based on their observation, they postulated that slow endoleak tends to resolve naturally within 1 year after TEVAR. Of 36 patients with type Ia endoleak, one died of aneurysm rupture and 7 resolved spontaneously indicating that active surveillance and timely treatment can lead to favorable results in selected cases (6, 9, 42).

The incidence of SCI is 4.12% in zone 0 TEVAR, near to that (4.5%) reported by Uchida, which included 7,309 patients treated by TEVAR in 2014 (64). There were no concrete explanations for SCI or risk factors in the reviewed studies. In theory, sacrifice of LSA inflow is a risk factor for SCI; however, absence of detailed information impeded analysis in this review. In this review, only one SCI patient had their LSA covered without revascularization (20). Nonetheless, previous studies have suggested that LSA revascularization in selected patients might prevent SCI (34). Authors also provided other preventive procedures including prophylactic cerebrospinal fluid drainage and maintenance of higher mean arterial pressure (18, 34). In this review, 8 cases of SCI were successfully treated with cerebrospinal fluid drainage. Only one case of permanent paraplegia was recorded, in a patient implanted with a long aortic stent, with LSA coverage (20).

The rate of post-operative RTAD in the present review is 5.72%, lower than that reported by Chen et al. in their meta-analysis. Their study, conducted in 2018, highlighted the higher risk of RTAD in zone 0 TEVAR compared to zones 1, 2, 3, and 4 (8.12 vs. 2.57, 2.66, 0.67,% and 0.67%, respectively) (65). The anatomy of the arch and lack of comfort with newer stent grafts might contribute to the high rate of RTAD in zone 0 TEVAR. In four reviewed studies, RTAD patients had undergone hybrid procedures, and the leading cause suggested by the authors was clamp injury while debranching or bypassing branch vessels (9, 21, 27, 46). Oversizing of the stent graft, a large diameter of the stent graft, and an acute angle formed by the ascending aorta and PLZ contribute to RTAD according to some authors (21, 51). Although previous studies suggested proximal bare-metal configuration as a risk factor for RTAD, this configuration was present in 60.56% of cases in the reviewed studies and did not correlate with the rate of RTAD (65, 66). Prevention of RTAD mainly focuses on pre-operative planning and stent graft design. Chassin-Trubert et al. (46) suggested that, in selected hybrid procedures, rapid right ventricular pacing might decrease the risk of RTAD following zone 0 TEVAR. Given the 4/17 (23.5%) related-death rate and 8/17 (47.0%) reintervention rate, surgeons should consider reintervention as soon as RTAD is discovered (6, 9, 15, 19, 21, 46).

In the present review, the overall 30-day/in-hospital death rate of zone 0 TEVAR is 7.49%, close to that reported for all zone TEVAR (8.07%) in a systematic review by Ramdass in 2015 (67). But it exceeds the rates after TEVAR for descending thoracic aortic disease, as shown by Naazie in 2022 and Harris in 2020 (4.2% in 2,141 patients and 4.0% in 1,784 patients, respectively) (68, 69). If arch disease is taken into account, the 30-day rate of death in this review is lower than that reported for frozen elephant trunk in a multicenter study by Leone (437 patients, 14.9%), and higher than that reported for open total arch replacement in a meta-analysis performed in 2016 (2,880 patients, 5.3%) (70, 71).

The present meta-analysis and systemic review shows some limitations. First, most studies were conducted in a single center and lacked specific clinical data on individual patients. Second, most of the reviewed studies focused on one or two techniques, leading significant reporting biases. Finally, the recruitment time in reviewed studies with high heterogeneity inhibited further analysis of yearly trends. Consequently, to obtain a complete picture of zone 0 TEVAR, more exhaustive investigations on zone 0 TEVAR are required in the future.

## 5. Conclusion

Despite the absence of stent grafts dedicated for zone 0 TEVAR, novel stent grafts and various techniques targeting zone 0 TEVAR are currently being investigated and developed. However, there is still no consensus on technique and device selection for zone 0 TEVAR in current practice. Furthermore, the post-operative stroke rate is relatively high, while other complications and perioperative death rate are comparable to those of TEVAR for other aortic zones. The small number of studies aimed at zone 0 TEVAR calls for more comprehensive and detailed clinical studies to improve the informed decision-making in patients who may benefit from zone 0 TEVAR.

## Data availability statement

The original contributions presented in the study are included in the article/supplementary material, further inquiries can be directed to the corresponding author.

## Author contributions

XL and LZ contributed to conception and design of the study, organized the data extraction and statistical analysis, and wrote sections of the manuscript. LZ wrote the first draft of the manuscript. All authors contributed to manuscript revision, read, and approved the submitted version.
